# Impact of HIV Comprehensive Care and Treatment on Serostatus Disclosure among Cameroonian Patients in Rural District Hospitals

**DOI:** 10.1371/journal.pone.0055225

**Published:** 2013-01-31

**Authors:** Marie Suzan-Monti, Charles Kouanfack, Sylvie Boyer, Jérôme Blanche, Renée-Cécile Bonono, Eric Delaporte, Patrizia M. Carrieri, Jean-Paul Moatti, Christian Laurent, Bruno Spire, Stratall ANRS 12-110/ESTHER Study Group

**Affiliations:** 1 INSERM, UMR912 (SESSTIM), Marseille, France; 2 Université Aix Marseille, UMR_S912, IRD, Marseille, France; 3 ORS PACA, Observatoire Régional de la Santé Provence Alpes Côte d’Azur, Marseille, France; 4 Central Hospital, UMI 233, Yaoundé, Cameroon; 5 Socio-anthropological Research Institute (IRSA), Catholic University of Central African States, Yaoundé, Cameroon; 6 Institut de Recherche pour le Développement (IRD), University Montpellier 1, UMI 233, Montpellier, France; 7 Department of Infectious and Tropical Diseases, University Hospital, Montpellier, France; Rollins School of Public Health, Emory University, United States of America

## Abstract

This work aimed to analyze the rate of disclosure to relatives and friends over time and to identify factors affecting disclosure among seropositive adults initiating antiretroviral therapy (ART) in rural district hospitals in the context of decentralized, integrated HIV care and task-shifting to nurses in Cameroon. Stratall was a 24-month, randomized, open-label trial comparing the effectiveness of clinical monitoring alone with laboratory plus clinical monitoring on treatment outcomes. It enrolled 459 HIV-infected ART-naive adults in 9 rural district hospitals in Cameroon. Participants in both groups were sometimes visited by nurses instead of physicians. Patients with complete data both at enrolment (M0) and at least at one follow-up visit were included in the present analysis. A mixed Poisson regression was used to estimate predictors of the evolution of disclosure index over 24 months (M24).The study population included 385 patients, accounting for 1733 face-to-face interviews at follow-up visits from M0 to M24. The median [IQR] number of categories of relatives and friends to whom patients had disclosed was 2 [1]–[3] and 3 [2]–[5] at M0 and M24 (p-trend<0.001), respectively. After multiple adjustments, factors associated with disclosure to a higher number of categories of relatives and friends were as follows: having revealed one’s status to one’s main partner, time on ART, HIV diagnosis during hospitalization, knowledge on ART and positive ratio of follow-up nurse-led to physician-led visits measuring task-shifting. ART delivered in the context of decentralized, integrated HIV care including task-shifting was associated with increased HIV serological status disclosure.

## Introduction

Scaling up access to HIV care and treatment worldwide has mainly been achieved in resource-limited countries by implementing the World Health Organization (WHO) public-health approach [1] of decentralized and integrated HIV care delivery. In order to overcome the shortage of healthcare staff, numerous African countries have developed different ART delivery models based on decentralization and task-shifting from physicians to nurses, community-based health workers and lay workers. As a result, those countries have seen a considerable increase in the number of HIV-positive patients receiving ART during the last ten years, especially in rural areas, and offer high-quality and cost-effective care [2], [3], [4].

Although several studies have been carried out to evaluate the impact of task-shifting on financial, structural and treatment outcomes, data on the impact of task-shifting on psychosocial outcomes remain scarce. Participants in one study on task-shifting, a home-based antiretroviral care program (HBAC) in rural Uganda monitored by lay workers, experienced positive social outcomes including family and community support together with relationship strengthening 3 months after enrolment [5], [6]. These positive outcomes were attributed to participation in the program. In Cameroon, support from HIV health care staff and task-shifting HIV care to nurses have been shown to be major structural correlates of patients’ adherence to ART in a national representative sample of people living with HIV (PLWH) participating in the ANRS 12-116 EVAL survey [5]. It is becoming increasingly evident that there is a need for comprehensive approaches to deliver HIV- and health-related services to PLWH, including psychosocial support and positive prevention interventions [6], [7].

Disclosure is a double-edged sword for seropositive people. On the one hand, it may expose to stigmatization and other negative social interactions [8] detrimental to PLWH’s psychosocial well-being [9]. On the other hand, it is considered to be a key component for positive prevention in PLWH in terms of reducing HIV transmission risk to sexual partners [10], especially in serodiscordant married or cohabiting couples, considered to be major contributors to the HIV/AIDS epidemics in sub-Saharan Africa [11], [12]. Disclosure is also a key component for treatment effectiveness: besides disclosure to one’s spouse/steady or casual sexual partner(s), disclosure to family members and friends is indeed necessary to ensure social and/or material support [13], [14], [15], [16] two major determinants of treatment adherence in resource-limited countries. To date, most analyses addressing this question have been performed in cross-sectional studies providing an overview of disclosure patterns at a given point in time. Among PLWH participating in the cross-sectional ANRS 12-116 EVAL survey in Cameroon, it was shown that access to ART encourages disclosure to relatives and friends [17]. Individual factors, access to psychosocial and economical support interventions were found to be associated with disclosure to one’s main partner [18]. Few studies have described the evolution of disclosure over time and particularly in resource-limited countries. In Mozambique, Pearson *et al*. reported that one year after ART initiation, disclosure to friends was associated with less stigma, compared to disclosure to family or to a partner [19]. In South Africa, Wouters *et al*. highlighted the positive, immediate and long term impact of community support on disclosure to family members [14]. However neither of these studies specifically investigated the rates and patterns of HIV status self-disclosure to family or friends relational over time.

In the present study we analyzed data collected from among PLWH initiating ART in the Stratall ANRS 12-110/ESTHER longitudinal trial in rural district hospitals [2] to: i) examine the rate of disclosure over 24 months for different categories of relationships including various immediate family members (father, mother, brother or sister, child), other family members and also friends; ii) characterize individual and/or structural factors associated with disclosure within the context of scaling up access to HIV care, a fundamental part of the national Cameroonian decentralized ART delivery program; iii) describe the impact of disclosure to one’s steady partner on disclosure to relatives and friends; iv) investigate the impact of exposure to task-shifting of HIV care from physicians to nurses on disclosure in the Cameroonian decentralized ART delivery program.

## Methods

### Study Population and Data Collection

Stratall was a 24-month, randomized, open-label trial which enrolled 459 HIV-infected ART-naive adults in 9 rural district hospitals in Cameroon over the period 2006–2010. The protocol was approved by the National Ethics Committee of Cameroon and the institutional Ethics Committee of the French Institut de Recherche pour le Développement (IRD). The trial was designed to compare the effectiveness of using a laboratory plus clinical (LAB) monitoring approach (six-monthly viral load and CD4 cell count) with clinical (CLIN) monitoring alone, when measuring ART effectiveness. Participants were recruited by hospital health-care workers during routine HIV care visits. They were eligible if they were 18 years or older, had confirmed HIV-1 group M infection, WHO clinical stage 3 or 4, or WHO clinical stage 2 with a total lymphocyte count of fewer than 1200 cells per µL. All participants provided written informed consent. They initiated ART at enrolment (month 0 (M0)). Face-to-face interviews were used to collect socio-demographic data and information about disclosure to relatives and friends as well as experience with HIV testing and care. Data regarding patients’ reasons for participating in the trial were also collected (e.g. free access to treatment and care, barriers to requesting financial support from relatives and friends associated with disclosure). The schedule of the medical visits and face-to-face interviews is shown in Table 1. Details on the full design and clinical results of the trial are described elsewhere [2]. Patients with complete data both at enrolment and at least at one follow-up visit were included in the present analysis (n = 385).

**Table 1:** Schedule of enrolment and follow-up visits.

	Enrolment	Follow-up visits									
	M0	Week2	M1	M3	M6	M9	M12	M15	M18	M21	M24
Visits in											
CLIN group	P^1^	N^1^	P	N	P	N	P	N	P	N	P
LAB group	P	P	P	P	P	P	P	P	P	P	P
ITW^2^	+		+	+	+		+		+		+
Disclosure assessment^3^	+			+	+		+				+

1 P: physician-led visit; N: nurse-led visit.

2 ITW: Follow-up points when face-to-face interviews took place.

3 Disclosure assessment: Follow-up points considered in the present analysis when disclosure was assessed during face-to-face interviews.

### HIV Disclosure Outcome Measures

At enrolment, M3, M6, M12 and M24, serological status disclosure to relatives and friends was evaluated during face-to-face interviews (n = 1733). Participants were asked: “Since finding out about being HIV-positive, who have you disclosed your seropositivity to?” Possible responses were: “1- father/2- mother/3- brother or sister/4- a child/5- other member of family/6- steady partner/7- others partners/8- friends/9- a priest”. These nine relationship types were analyzed as categories, such that each positive answer for each category received a maximum value of “1″, independently of the number of persons to whom the participant had disclosed to in that category and irrespective of any change in their composition after disclosure. For example, even if a participant had disclosed to more than one ‘other family member’ or ‘friend’, only the value 1 would be attributed to these categories. The main outcome was a **cumulative disclosure index**, ranging from 0 to 6, computed at each time point after ART initiation as the sum of six categories to whom patients had disclosed their serological status up to that point in time, as follows: father, mother, brother or sister, child, other family members and friends. As a cumulative measure, this index could only remain stable or increase over time. Disclosure to “priest” was not included in the disclosure index since the present analysis was focused on family and friends networks. The categories “steady partner” and “others partners” were not included into the disclosure index either, since only 180 (46.7%) study participants had a steady partner and among these a high percentage (70%) had already revealed their serostatus to their steady partners at enrolment. Disclosure to one’s steady partner was a variable considered as a potential correlate and its association with disclosure to relatives and friends was investigated.

An **overall rate of disclosure** (% disclosure in the study sample) was calculated at each follow-up time point (M3, M6, M12, M24) as the percentage of participants having revealed their serostatus to each of the six relationship categories.

### Potential Correlates of the Outcome

To identify correlates of serostatus disclosure over time the following sets of participants- and health care-related variables were considered and their association with the outcome explored.

#### Socio-demographic variables

Age, gender, educational level, marital status, having a steady partner, being the head of the household, employment, and time taken to reach district hospital.

#### Clinical characteristics

WHO clinical stage, CD4 cell count and viral load at enrolment.

#### Experience of hospitalization since HIV diagnosis

Participants were asked whether they had been hospitalized, for at least one night since finding out about their HIV status (yes/no).

#### Confidence in HIV health care staff

Participants were asked whether they felt very confident, not very confident or not at all confident about the quality of care provided by HIV medical staff.

#### HIV testing during hospitalization

At enrolment, patients were asked under which circumstances they had initially been tested for HIV. Possible answers were categorized as follows: a) during hospitalization or b) other circumstances (e.g. because of clinical symptoms, tuberculosis, pregnancy, blood donation or routine medical consultation; because of main partner’s seropositivity, a new relationship with a new partner, a testing campaign, TV and media advertisements, relatives’ advice etc.).

#### Belief that ART can cure HIV

Patients were asked the following question at 3 follow-up points (M0, M12, and M24): “Do you think ART can cure HIV?” The answers “yes” or “I don’t know” were considered incorrect whereas the answer “no” was considered correct.

#### Ratio of nurse-led to physician-led visits

At enrolment, patients were randomly assigned to clinical monitoring alone (CLIN) or laboratory plus clinical monitoring (LAB) and referred to a physician. Follow-up visits were scheduled at week 2, M1, M3 and every 3 months thereafter until M24 (Table 1). Of the 10 follow-up visits planned for the CLIN participants, 5 were supposed to be task-shifted to nurses, physicians performing the others. All 10 follow-up visits planned for the LAB participants were supposed to be performed by physicians. In reality, because of various problems, including the unavailability of physicians and local healthcare organization difficulties, certain LAB participants were sometimes seen by nurses instead of physicians and vice versa for clinically monitored participants. In order to take this contamination into account, the level of exposure to task-shifting was evaluated as the ratio of total really nurse-led visits to total really physician-led visits calculated at each follow-up time point (M3, M6, M12, M24).

### Statistical Analysis

As the outcome was the cumulative index of total number categories of relatives and friends to whom individuals had disclosed their serostatus at each follow-up point, mixed Poisson regression models were used to identify independent correlates of the outcome and estimate incidence rate ratios (IRR) and their 95% confidence intervals. This mixed model approach takes into account the repeated measure design and is currently considered as the standard approach for analyzing repeated measure design where the outcome is a count or a continuous variable [20]. Factors associated with disclosure with a p value <0.25 in the univariate analysis were introduced into the multivariate analysis. A stepwise procedure was then used to select significant factors (entry threshold p value <0.05). Since all participants initiated ART at enrolment, “Time on ART” was included in the model as a time-varying covariate for the outcome, measured at M3, M6, M12 and M24. Additionally, a mixed Poisson regression model was also used in the univariate analysis to measure the impact of disclosure to one’s steady partner on disclosure to relatives and friends. Analyses were performed using Stata version 10.0 (Stata Corporation, College Station, Texas, USA) and SPSS version 18.0 (SPSS Inc, Chicago, Illinois, USA).

## Results

### Participants’ Characteristics

Among the 385 participants included in the present analysis, 184 and 201 were randomized in the LAB and CLIN groups respectively. Socio-demographic characteristics of the participants are described in Table 2. At enrolment, median [IQR] age was 36 years [30–44], 71.4% of participants were female, 52.5% were single, 56.6% were in active employment and 41% were heads of their household. A minority of participants (16.5%) were HIV-diagnosed during hospitalization. Clinical data showed that 51% of participants had a CD4 cell count <200/mm3 and almost all participants were at WHO clinical stage 3 (75.1%) or 4 (24.6%). At enrolment, median [IIQ] time since HIV diagnosis was 3 [1]–[9] months. There were no significant differences in the socio-demographic characteristics of participants in the LAB or CLIN group.

**Table 2:** Baseline characteristics of participants (N = 385).

	N (%) or median [IQR]
Gender	
-Women	275 (71.4)
-Men	110 (28.6)
Age (years)	36 [30–44]
Educational level	
-Lower than secondary school	189 (49.1)
-Secondary school or higher	196 (50.9)
Marital status	
-Married or cohabiting	121 (31.4)
-Divorced or separate	11 (2.9)
-Widowed	51 (13.2)
-Single	202 (52.5)
Head of the household	
-No	222 (59)
-Yes	154 (41)
Active employment	
-No	150 (43.4)
-Yes	196 (56.6)
Time taken to reach district hospital	
-Less than 30 mins	172 (45.6)
-Between 30 mins and 1 hour	116 (30.8)
-Between 1 and 2 hours	61 (16.2)
-More than 2 hours	28 (7.4)
Circumstances of HIV testing	
-During hospitalization	62 (16.5)
-Other	314 (83.5)
WHO clinical stage	
−2	1 (0.3)
−3	289 (75.1)
−4	95 (24.6)
CD4 cell count (cells/µL)	196 [94–358]
Viral load (log_10_ copies/mL)	5.6 [5.2–6.0]

Participants accounted for 1733 follow-up visits from enrolment to M24, 900 and 833 in the LAB and CLIN groups respectively. Overall, 483 (28%) visits were task-shifted to nurses. The ratio of visits led by nurses/visits led by physicians was 0 [0–0.1] for the LAB group and 0.3 [0–0.7] for the CLIN group. Among the 385 participants in the study sample, 27 (7%) were considered as lost to follow-up, 11 (6%) and 16 (8%) from the LAB and CLIN groups respectively. However since these participants had complete data at M0 and at one follow-up visit, they were considered in the present analysis. Interestingly, for those participants lost to follow-up in the present analysis, only 12 out of 83 follow-up visits were task-shifted to nurses (14%).

Fifty five PLWH enrolled in the Stratall trial were not included in the present analysis since they had complete data only at enrolment (M0). Socio-demographic characteristics for these 55 were not significantly different from those of the PLWH who were included, in terms of gender, age, marital status, being the head of their household and CD4 cell count (data not shown). However, a significantly higher proportion of these 55 were unemployed (n = 10 (22.2%), p<0.001).

### Rates of Disclosure Over Time

In the study sample, a majority of participants (n = 313, 81.3%) increased their overall rate of disclosure over time from M0 to M24, irrespective of the six possible relationship categories (father, mother etc.) assessed in the present analysis. Among the 72 PLWH whose disclosure index remained constant over time, 14 did not reveal their serostatus to anyone (3.6% of the study sample).

**Figure 1:**
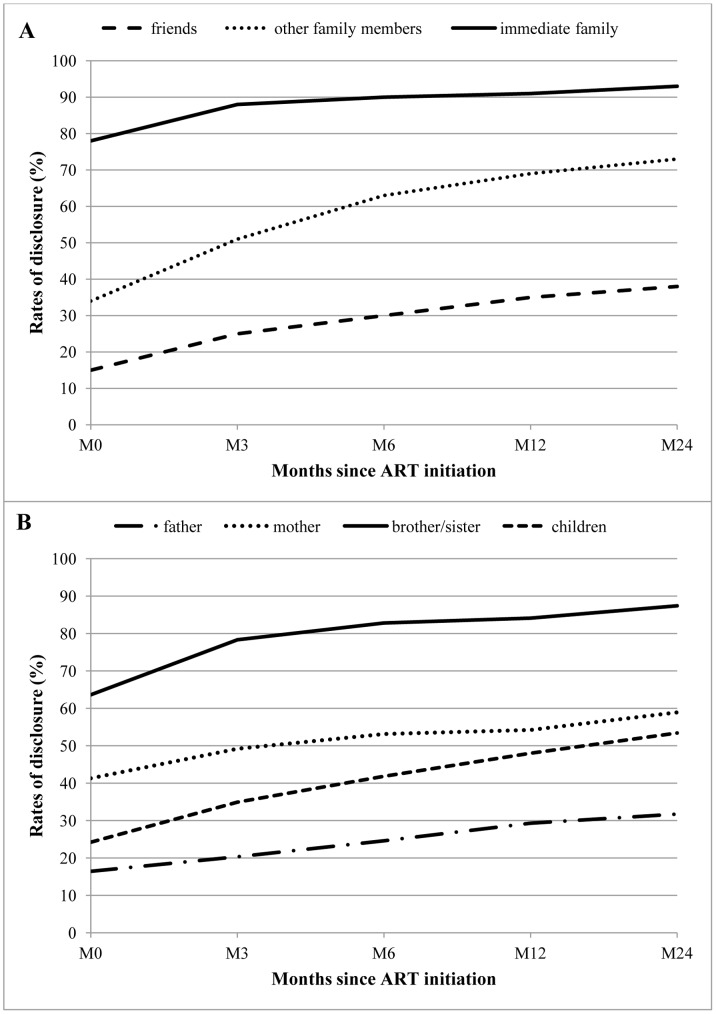
Overall rate of disclosure over time since initiating ART. The overall rate of disclosure to different categories of relatives and friends (panel A) and to different members among immediate family (panel B) was calculated at enrolment (M0) and at each follow-up time point (M3, M6, M12, M24) as the percentage of participants having revealed their serostatus to each of the relationship categories.

When considering the different categories of people with whom PLWH disclosed their serostatus, the overall rate of disclosure to one or more immediate family member categories (father, mother, brother, sister, child) ranged from 78% at enrolment to 93% at M24, 34% to 73% for disclosure to the “other family members” category and 15% to 38% to “friends” (Figure 1A). Among the immediate family members, the overall rate increased over time from 64% to 87% for disclosure to siblings, 41% to 59% for mothers, 24% to 53% for children and 16% to 32% for fathers (Figure 1B).

The median [IQR] number of categories of relatives and friends to whom patients had disclosed (i.e. be they from immediate family categories, “other family members” or “friends”) was 2 [1]–[3] and 3 [2]–[5] at M0 and M24 (p-trend<0.001), respectively.

### Factors Associated with the HIV Disclosure Index

The following factors did not appear to be significantly associated with disclosure (Table 3): gender, educational level, being in active employment, and randomization group (LAB versus CLIN). Variables with a p-value <0.05 in the univariate analysis which were not significant in the multivariate analysis are as follows: having a main partner (IRR [95%CI] 1.1 [1.0–1.2], p = 0.03), time to reach the district hospital >2 hours (IRR [95%CI] 1.2 [1.1–1.3], p = 0.002), experience of hospitalization (IRR [95%CI] 0.9 [0.8–1.0], p = 0.003) and number of visits led either by physicians (IRR [95%CI] 1.1 [1.1–1.1], p<0.001) or by nurses (IRR [95%CI] 1.1 [1.08–1.1], p<0.001).

**Table 3:** Factors associated with HIV serostatus disclosure to relatives (Mixed Poisson regression model N = 385).

	Visits	Univariate analysis		Multivariate analysis		
	N (%) or median [IQR]	IRR (95%CI)	p-value	IRR (95%CI)	p-value	
Gender						
-Women	1243 (71.7)					
-Men	490 (28.3)	0.9 [0.8; 1.1]	0.42			
Educational level						
-Lower than secondary school	852 (49.2)					
-Secondary school or higher	881 (50.8)	0.9 [0.8; 1.1]	0.31			
Active employment						
-No	622 (37)					
-Yes	1059 (63)	1.0 [0.9; 1.1]	0.27			
Study group						
-CLIN	900 (51.9)					
-LAB	833 (48.1)	1.0 [0.9; 1.1]	0.97			
Having a steady partner						
-Yes	925 (53.4)	1.1 [1.0–1.2]				
-No	808 (46.6)	1	0.03			
Head of the household						
-No	979 (56.8)	1				
-Yes	745 (43.2)	0.9 [0.9–1.0]	0.23			
Time taken to reach district hospital						
-Less than 30 mins	616 (37.3)	1				
-Between 30 mins and 1 hour	476 (28.8)	1.0 [0.9–1.1]	0.45			
-Between 1 and 2 hours	311 (18.8)	1.1 [1.0–1.2]	0.16			
-More than 2 hours	248 (15)	1.2 [1.1–1.3]	0.002			
Circumstances of HIV testing						
-During hospitalization	276 (16.3)	1.2 [1.0–1.4]		1.2 [1.0–1.4]		
-Other	1417 (83.7)	1	0.03	1	0.02	
Time on ART						
-Enrolment (M0)	385 (22.2)	1		1		
-Follow-up (until M24)	1348 (77.8)	1.5 [1.4–1.7]	<0.001	1.5 [1.3–1.6]	<0.001	
Experience of hospitalization since HIV diagnosis						
-No	1490 (86.9)	1				
-Yes	225 (13.1)	0.9 [0.8–1.0]	0.003			
Belief that ART can cure HIV						
-No	492 (28.7)	1		1		
-Yes	1223 (71.3)	0.9 [0.8–0.9]	0.001	0.9 [0.8–1.0]	0.03	
Confidence in HIV healthcare staff						
-No	104 (6.1)	1				
-Yes	1596 (93.9)	1.1 [1.0–1.3]	0.10			
Number of visits led by (per 1-visit increment)						
-Physician	4 [2]–[6]	1.1 [1.1–1.1]	<0.001			
-Nurse	1 [0–2]	1.1 [1.08–1.1]	<0.001			
Ratio of nurse-led to physician-led visits (per 1-unit increment)	0.1 [0.0–0.4]	1.5 [1.4–1.7]	<0.001	.1 [1.0–1.3]	0.05	

Among these individual factors, we analyzed the impact of disclosure to one’s steady partner on disclosure to relatives (i.e. immediate family or other family members) and friends. Among the 180 study sample participants who had a steady partner at enrolment, 70% (N = 126) had revealed their serological status to them. Disclosure to one’s steady partner was associated with disclosure to a higher number of relatives and friends (IRR = 1.25 [1.1; 1.5], p = 0.002). We found no significant difference for disclosure to relatives and friends between patients who, at enrolment, did not have a steady partner (N = 205) and those who did have one but had not disclosed their serological status to them (N = 41; IRR = 1.00 [0.9; 1.1], p = 0.93).

The multivariate analysis showed that the following factors significantly associated with serological status disclosure to a higher number of categories of relatives and friends were: being HIV-diagnosed during hospitalization (IRR [95%CI] 1.2 [1.0–1.4], p = 0.02), time on ART (IRR [95%CI] 1.5 [1.3–1.6], p<0.001), and having a positive ratio of nurse-led to physician-led visits (IRR [95%CI] 1.1 [1.0–1.3], p = 0.05). On the contrary, participants who believed that ART could cure HIV were more likely to conceal their seropositivity from their relatives and friends (IRR [95%CI] 0.9 [0.8–1.0], p = 0.03).

## Discussion

The analysis of longitudinal data collected during the Stratall ANRS 12-110/ESTHER trial allowed us to show that patients having been HIV-diagnosed during hospitalization and initiating ART in rural district hospitals in Cameroon were more likely to disclose their seropositivity to a higher number of relatives and friends than those diagnosed under other circumstances. Furthermore, those believing that ART cures HIV tended to conceal their serostatus more than those who did not think so. Disclosure to one’s steady partner was positively associated with a higher rate of disclosure to relatives and friends. In addition, this study showed that the positive ratio of nurse-led to physician-led follow-up visits was significantly associated with a higher disclosure index.

The present analysis, nested inside the Stratall ANRS 12-110/ESTHER trial, was based on longitudinal socio-behavioral data collection. It allowed us to investigate the rates of patient self-disclosure to relatives and friends over a follow-up period of 2 years. However, the data collected did not enable us to investigate PLWH disclosure to other categories of people or whether their status was revealed by someone else. Disclosure is known to be a correlate of safer sexual practices [10], [21], [22] and better adherence to ART [23], [24], and as such must be encouraged to assure improved treatment response. We previously addressed the question of disclosure to the main partner or to relatives and friends using data from the cross-sectional EVAL ANRS 12-116 survey in Cameroon [17], [18] which included HIV-positive individuals receiving ART or not. That survey was the first to document the effect of access to ART on a set of psychosocial outcomes within a given population in a specific resource-limited setting. In the present analysis, data were collected from among HIV-positive people initiating ART at study enrolment. The results reported here are complementary to the ones mentioned just above [17], [18] and provide an original longitudinal assessment of this topic within the context of a Sub-Saharan country that has developed a large national program for access to ART based on the decentralization of HIV care.

To date, most published studies describing disclosure to family members, friends, partners, colleagues and health workers in Sub-Saharan countries have been based on cross-sectional surveys [25], [26], [27]. Longitudinal studies of disclosure to different types of relationships are scarce. Some have been carried out in Midwestern USA on men who have sex with men [28] and on women [29], others have been carried out in general populations in various African countries including South Africa [14], [30], Zimbabwe [31], and Ethiopia [32]. The present analysis highlighted that study participants revealed their serological status most often to their immediate family members (primarily to their siblings and then to their mother), then to other family members and lastly to friends. One limitation of this analysis is that the different categories of relationships were not considered in the primary analysis of factors affecting disclosure since, according to the category of relatives and friends to whom participants disclosed to, the sample would have been not sufficient to allow a powerful analysis. The present results are in accordance with those of previous studies [15], [18], [25], [32]. Furthermore, as was the case for one cross-sectional study in Ethiopia [33], participant gender was not found to be significant in regard to disclosure. Since our study sample was composed of HIV-positive people at advanced stages of the disease, disclosing one’s status to parents and siblings may have been the best way to look for emotional or economical support (e.g. health expenditures) or care [14], [16], [34], [35].

Revealing one’s seropositivity to one’s steady partner was identified as a positive predictive individual factor of future disclosure to relatives and friends. Rates of disclosure to steady partners varied widely from about 20% to about 91% depending on the study, population and setting investigated [16], [18], [19], [22], [25], [26], [27], [30], [31], [33]. Unlike the present study however, none of these analyses reported an association between disclosure to the steady partner and disclosure to other relationship categories. One possible explanation for the result found here is that although disclosure to one’s steady partner exposes PLWH to the risk of rejection, it may nonetheless encourage support from this most intimate relationship, thereby setting the stage for disclosure to other relatives and friends. Furthermore, such disclosure can improve PLWHs well-being and reduce HIV transmission.

Our study evaluated the number of different categories of people to whom PLWHs disclosed their serostatus. Although one cannot suppose that in general patients disclose their status to everyone, one possible limitation of this analysis could be that patients may not have had the same number of potential people to disclose their seropositivity to. While it was not feasible in this analysis to measure the potential number of people to whom patients could reveal their serostatus, it is important to note that a large majority of participants (>80%) increased their cumulative general index over time and 96.4% of the study sample revealed their seropositivity at least to one person. These figures were similar to those found in a study in Ethiopia [32]. Various rates of disclosure among HIV-positive adults have been reported for various populations, cultures and settings investigated [34], [36]. Those we observed in Cameroon (in the present analysis and in [17], [18], [22]) were among the highest reported to date and may be the result of the scaling-up access to ART based on a program of decentralized HIV care which includes psycho-social support [37]. Although our study provides another example of the “selective” pattern of gradual disclosure to a growing number of people [13], [36], the initial objective and quantitative nature of the trial did not allow us to document decisions, reactions or reasons for disclosure, which can be better explored in qualitative studies. Further longitudinal studies focusing on this specific issue are necessary to provide a better estimate of overall disclosure to different members of one’s family and one’s friends.

HIV diagnosis during hospitalization stood out as being significantly associated with disclosure to relatives and friends, irrespective of who initiated the test (patient or provider). In our study sample, a minority of participants were HIV-diagnosed during hospitalization, while others were tested positive in outpatient care. HIV diagnosis during hospitalization in comprehensive care facilities may allow earlier referral of patients to support initiatives carried out by nurses [8] or community health workers [14], [35]. In 2007, in order to expand the practice of voluntary counseling and testing, the WHO recommended healthcare provider-initiated testing as a standard component of medical care in settings with generalized HIV epidemics [38] with the belief that it would encourage more timely HIV care and support interventions. Indeed, one subsequent study in rural Ugandan areas implementing such provider-initiated testing highlighted a high rate of disclosure in outpatients receiving routine testing [39].

In the present study misunderstanding medical information was shown to be a factor significantly associated with serostatus concealment. In our study sample -where all participants initiated ART at enrolment- 71% of them thought that ART could cure HIV. PLWHs might delay disclosure to their family and friends in the hope that ART will cure them. In the EVAL ANRS 12-116 survey in Cameroon [22] misguided beliefs about the benefits of ART were seen to have deleterious effects on disclosure to one’s steady partner in HIV-positive women. Positive prevention strategies must deliver accurate medical information to HIV-positive patients in order to limit possible patient misunderstanding and overestimation of ART effectiveness.

The most common environmental factors, such as interruptions of supply of antiretroviral treatment (“stock outs”), and characteristics related to models of HIV treatment delivery, such as task-shifting HIV care to nurses, were studied as possible correlates of ART adherence or interruption [5]. Most studies evaluating task-shifting as an environmental factor or a model-of-care component have focused on efficiency and increased access to ART as well as affordability, quality of care, health outcomes and team dynamics [3], [4]. The present study is the first to highlight an association between a factor related to the model-of-care and disclosure to one’s partners, family and friends. The positive impact on disclosure of task-shifting follow-up visits to nurses might be due to the closer proximity patients have with nurses than with physicians. Despite the fact that all medical staff involved in ART programs have heavy workloads, nurses may be more readily available than physicians to listen to patients’ concerns about HIV disease and ART treatment management [40]. In Cameroon, task-shifting was introduced as part of the national ART program launched in 2001 [41] and free-access to ART was adopted in May 2007. The decentralization of ART delivery programs is a major issue for public health policies in resource-limited countries, due to both the large number of PLWH who need ART and to severe healthcare staff shortages. The WHO global health sector strategy 2011–2015 recommends building strong and sustainable systems [42]. Task-shifting from highly-trained to both lesser-trained professional health workers and community-based health workers is part of the response to this challenge [43].

In conclusion, our results pointed out that ART delivered in the context of decentralized, integrated HIV care including task-shifting was associated with increased HIV serological status disclosure. Task-shifting to nurses must be promoted not only as a means to overcome shortages in physician numbers and to improve workload sharing among facilities and healthcare workers, but also to provide PLWH with an environment which is more understanding and which favors long-term ART success and psychosocial well-being.

## References

[1] World Health Organization (2010) Towards universal access. Scaling up priority HIV/AIDS interventions in the health sector. Available : http://whqlibdoc.who.int/publications/2010/9789241500395_eng.pdf. Accessed 2013 Jan 3.

[2] Laurent C, Kouanfack C, Laborde-Balen G, Aghokeng AF, Mbougua JB, et al (2011) Monitoring of HIV viral loads, CD4 cell counts, and clinical assessments versus clinical monitoring alone for antiretroviral therapy in rural district hospitals in Cameroon (Stratall ANRS 12110/ESTHER): a randomised non-inferiority trial. Lancet Infect Dis 11: 825–833. 21831714 10.1016/S1473-3099(11)70168-2

[3] Callaghan M, Ford N, Schneider H (2010) A systematic review of task- shifting for HIV treatment and care in Africa. Hum Resour Health 8: 8. 20356363 10.1186/1478-4491-8-8 PMC2873343

[4] Assefa Y, Van Damme W, Hermann K (2010) Human resource aspects of antiretroviral treatment delivery models: current practices and recommendations. Curr Opin HIV AIDS 5: 78–82. 20046151 10.1097/COH.0b013e328333b87a

[5] Boyer S, Clerc I, Bonono CR, Marcellin F, Bile PC, et al (2011) Non-adherence to antiretroviral treatment and unplanned treatment interruption among people living with HIV/AIDS in Cameroon: Individual and healthcare supply-related factors. Soc Sci Med 72: 1383–1392. 21470734 10.1016/j.socscimed.2011.02.030

[6] Bunnell R, Mermin J, De Cock K (2006) HIV prevention for a threatened continent. Implementing positive prevention in Africa. Journal of American Medical Association 296: 855–858. 10.1001/jama.296.7.855 16905790

[7] Matovu JK (2010) Preventing HIV transmission in married and cohabiting HIV-discordant couples in sub-Saharan Africa through combination prevention. Curr HIV Res 8: 430–440. 20636280 10.2174/157016210793499303

[8] Black BP, Miles MS (2002) Calculating the risks and benefits of disclosure in African American women who have HIV. J Obstet Gynecol Neonatal Nurs 31: 688–697. 10.1177/0884217502239211 12465865

[9] Stutterheim SE, Pryor JB, Bos AE, Hoogendijk R, Muris P, et al (2009) HIV-related stigma and psychological distress: the harmful effects of specific stigma manifestations in various social settings. AIDS 23: 2353–2357. 19741478 10.1097/QAD.0b013e3283320dce

[10] Pinkerton SD, Galletly CL (2007) Reducing HIV transmission risk by increasing serostatus disclosure: a mathematical modeling analysis. AIDS Behav 11: 698–705. 17082982 10.1007/s10461-006-9187-2 PMC2408867

[11] Eyawo O, de Walque D, Ford N, Gakii G, Lester RT, et al (2010) HIV status in discordant couples in sub-Saharan Africa: a systematic review and meta-analysis. Lancet Infect Dis 10: 770–777. 20926347 10.1016/S1473-3099(10)70189-4

[12] Guthrie BL, de Bruyn G, Farquhar C (2007) HIV-1-discordant couples in sub-Saharan Africa: explanations and implications for high rates of discordancy. Curr HIV Res 5: 416–429. 17627505 10.2174/157016207781023992

[13] Kalichman SC, DiMarco M, Austin J, Luke W, DiFonzo K (2003) Stress, social support, and HIV-status disclosure to family and friends among HIV-positive men and women. J Behav Med 26: 315–332. 12921006 10.1023/a:1024252926930

[14] Wouters E, van Loon F, van Rensburg D, Meulemans H (2009) Community support and disclosure of HIV serostatus to family members by public-sector antiretroviral treatment patients in the Free State Province of South Africa. AIDS Patient Care STDS 23: 357–364. 19327099 10.1089/apc.2008.0201

[15] Wong LH, Rooyen HV, Modiba P, Richter L, Gray G, et al (2009) Test and tell: correlates and consequences of testing and disclosure of HIV status in South Africa (HPTN 043 Project Accept). J Acquir Immune Defic Syndr 50: 215–222. 19131885 10.1097/QAI.0b013e3181900172 PMC2729272

[16] Ssali SN, Atuyambe L, Tumwine C, Segujja E, Nekesa N, et al (2010) Reasons for disclosure of HIV status by people living with HIV/AIDS and in HIV care in Uganda: an exploratory study. AIDS Patient Care STDS 24: 675–681. 20863244 10.1089/apc.2010.0062 PMC3826576

[17] Marcellin F, Bonono CR, Blanche J, Carrieri MP, Spire B, et al (2010) Higher risk of unsafe sex and impaired quality of life among patients not receiving antiretroviral therapy in Cameroon: results from the EVAL survey (ANRS 12–116). AIDS 24 Suppl 1 S17–25. 20023436 10.1097/01.aids.0000366079.83568.a2

[18] Suzan-Monti M, Blanche J, Bilé P, Koulla-Shiro S, Abu-Zaineh M, et al (2011) Individual and structural factors associated with HIV status disclosure to main partner in Cameroon: ANRS 12–116 EVAL survey, 2006–2007. J Acquir Immune Defic Syndr 57 Suppl (1) S22–S26. 10.1097/QAI.0b013e31821fcfa8 21857281

[19] Pearson CR, Micek MA, Pfeiffer J, Montoya P, Matediane E, et al (2009) One year after ART initiation: psychosocial factors associated with stigma among HIV-positive Mozambicans. AIDS Behav 13: 1189–1196. 19639405 10.1007/s10461-009-9596-0 PMC2901423

[20] Burton P, Gurrin L, Sly P (1998) Extending the simple linear regression model to account for correlated responses: an introduction to generalized estimating equations and multi-level mixed modelling. Stat Med 17: 1261–1291. 9670414 10.1002/(sici)1097-0258(19980615)17:11<1261::aid-sim846>3.0.co;2-z

[21] Crepaz N, Marks G (2003) Serostatus disclosure, sexual communication and safer sex in HIV-positive men. AIDS Care 15: 379–387. 12745398 10.1080/0954012031000105432

[22] Loubiere S, Peretti-Watel P, Boyer S, Blanche J, Abega SC, et al (2009) HIV disclosure and unsafe sex among HIV-infected women in Cameroon: results from the ANRS-EVAL study. Soc Sci Med 69: 885–891. 19560244 10.1016/j.socscimed.2009.05.044

[23] Peretti-Watel P, Spire B, Pierret J, Lert F, Obadia Y (2006) Management of HIV-related stigma and adherence to HAART: evidence from a large representative sample of outpatients attending French hospitals (ANRS-EN12-VESPA 2003). AIDS Care 18: 254–261. 16546787 10.1080/09540120500456193

[24] Do NT, Phiri K, Bussmann H, Gaolathe T, Marlink RG, et al (2010) Psychosocial factors affecting medication adherence among HIV-1 infected adults receiving combination antiretroviral therapy (cART) in Botswana. AIDS Res Hum Retroviruses 26: 685–691. 20518649 10.1089/aid.2009.0222 PMC4056458

[25] Akani CI, Erhabor O (2006) Rate, pattern and barriers of HIV serostatus disclosure in a resource-limited setting in the Niger delta of Nigeria. Trop Doct 36: 87–89. 16611440 10.1258/004947506776593378

[26] Antelman G, Smith Fawzi MC, Kaaya S, Mbwambo J, Msamanga GI, et al (2001) Predictors of HIV-1 serostatus disclosure: a prospective study among HIV-infected pregnant women in Dar es Salaam, Tanzania. AIDS 15: 1865–1874. 11579250 10.1097/00002030-200109280-00017 PMC6261328

[27] King R, Katuntu D, Lifshay J, Packel L, Batamwita R, et al (2008) Processes and outcomes of HIV serostatus disclosure to sexual partners among people living with HIV in Uganda. AIDS Behav 12: 232–243. 17828450 10.1007/s10461-007-9307-7

[28] Serovich JM, Esbensen AJ, Mason TL (2007) Disclosure of positive HIV serostatus by men who have sex with men to family and friends over time. AIDS Patient Care STDS 21: 492–500. 17651030 10.1089/apc.2005.0002 PMC1986738

[29] Serovich JM, Craft SM, Yoon HJ (2007) Women’s HIV disclosure to immediate family. AIDS Patient Care STDS 21: 970–980. 18154493 10.1089/apc.2007.0038 PMC2189577

[30] Simbayi LC, Kalichman SC, Strebel A, Cloete A, Henda N, et al (2007) Disclosure of HIV status to sex partners and sexual risk behaviours among HIV-positive men and women, Cape Town, South Africa. Sex Transm Infect 83: 29–34. 16790562 10.1136/sti.2006.019893 PMC2598581

[31] Kangwende RA, Chirenda J, Mudyiradima RF (2009) HIV status disclosure among people living with HIV/AIDS at FASO, Mutare, Zimbabwe. Cent Afr J Med 55: 1–7. 21977839 10.4314/cajm.v55i1-4.63632

[32] Deribe K, Woldemichael K, Wondafrash M, Haile A, Amberbir A (2008) Disclosure experience and associated factors among HIV positive men and women clinical service users in Southwest Ethiopia. BMC Public Health 8: 81. 18312653 10.1186/1471-2458-8-81 PMC2275263

[33] Deribe K, Woldemichael K, Bernard N, Yakob B (2009) Gender difference in HIV status disclosure among HIV positive service users. East Afr J Public Health 6: 248–255. 20803914

[34] Mayfield Arnold E, Rice E, Flannery D, Rotheram-Borus MJ (2008) HIV disclosure among adults living with HIV. AIDS Care 20: 80–92. 18278618 10.1080/09540120701449138

[35] Wouters E, Van Damme W, Van Loon F, van Rensburg D, Meulemans H (2009) Public-sector ART in the Free State Province, South Africa: community support as an important determinant of outcome. Soc Sci Med 69: 1177–1185. 19692165 10.1016/j.socscimed.2009.07.034

[36] Obermeyer CM, Baijal P, Pegurri E (2011) Facilitating HIV disclosure across diverse settings: a review. Am J Public Health 101: 1011–1023. 21493947 10.2105/AJPH.2010.300102 PMC3093267

[37] Boyer S, Protopopescu C, Marcellin F, Carrieri MP, Koulla-Shiro S, et al (2012) Performance of HIV care decentralization from the patient’s perspective: health-related quality of life and perceived quality of services in Cameroon. Health Policy Plan 27: 301–315. 21690123 10.1093/heapol/czr039

[38] World Health Organization (2007) Guidance on provider-initiated HIV testing and counseling in health facilities. Available : http://whqlibdoc.who.int/publications/2007/9789241595568_eng.pdf. Accessed 2013 Jan 3.

[39] Kiene SM, Bateganya M, Wanyenze R, Lule H, Nantaba H, et al (2010) Initial outcomes of provider-initiated routine HIV testing and counseling during outpatient care at a rural Ugandan hospital: risky sexual behavior, partner HIV testing, disclosure, and HIV care seeking. AIDS Patient Care STDS 24: 117–126. 20059356 10.1089/apc.2009.0269 PMC2835392

[40] Laurent C (2011) Commentary: scaling up HIV treatment in resource-limited countries: the challenge of staff shortages. J Public Health Policy 32: 211–218. 21346787 10.1057/jphp.2011.8

[41] Ministère de la Santé Publique (2008) Vers l’accès universel au traitement et à la prise en charge du VIH/Sida chez les adultes et les enfants au Cameroun [in French]. Yaoundé: Ministère de la Santé Publique and Comité National de Lutte contre le VIH/Sida du Cameroun.

[42] World Health Organization (2011) Global health sector strategy on HIV/AIDS 2011–2015. Available : http://whqlibdoc.who.int/publications/2011/9789241501651_eng.pdf. Accessed 2013 Jan 3.

[43] Philips M, Zachariah R, Venis S (2008) Task shifting for antiretroviral treatment delivery in sub-Saharan Africa: not a panacea. Lancet 371: 682–684. 18295026 10.1016/S0140-6736(08)60307-4

