# Correction: Impact of HIV comprehensive care and treatment on serostatus disclosure among Cameroonian patients in rural district hospitals

**DOI:** 10.1371/journal.pone.0323691

**Published:** 2025-05-08

**Authors:** Marie Suzan-Monti, Charles Kouanfack, Sylvie Boyer, Jérôme Blanche, Renée-Cécile Bonono, Eric Delaporte, Patrizia M. Carrieri, Jean-Paul Moatti, Christian Laurent, Bruno Spire, Stratall ANRS 12-110/ESTHER Study Group

The name of a member of the Stratall ANRS 12-110/ESTHER Study Group was incorrectly listed as J. Fokom. The correct name is Joël Fokom Domgue.

The corrected Acknowledgments should read: We thank all the patients and staff of the district hospitals who participated in the study. We would also like to thank Jude Sweeney for revising and editing the English version of the manuscript. This work was presented at the AIDS Impact meeting, Santa Fe (USA), 12–15 September 2011.

**Membership of the Stratall ANRS 12-110/ESTHER Study Group:** M. Biwolé-Sida, C. Kouanfack, S. Koulla-Shiro (Central hospital, Yaoundé, Cameroon); A. Bourgeois, E. Delaporte, C. Laurent, M. Peeters (IRD, University Montpellier 1, UMI 233, Montpellier, France); G. Laborde-Balen (French Ministry of Foreign Affairs, Yaoundé, Cameroon); M. Dontsop, S. Kazé, J-M. Mben (IRD, Yaoundé, Cameroon); A. Aghokeng, M.G. Edoul, E. Mpoudi-Ngolé, M. Tongo (Virology Laboratory, IMPM/CREMER/IRD-UMI 233, Yaoundé, Cameroon); S. Boyer, M.P. Carrieri, F. Marcellin, J-P. Moatti, B. Spire (INSERM, IRD, University Marseille, UMR 912, Marseille, France); C. Abé, S-C. Abega, C-R. Bonono, H. Mimcheu, S. Ngo Yebga, C. Paul Bile (IRSA, Catholic University of Central Africa, Yaoundé, Cameroon); S. Abada, T. Abanda, J. Baga, P. Bilobi Fouda, P. Etong Mve, G. Fetse Tama, H. Kemo, A. Ongodo, V. Tadewa, HD. Voundi (District Hospital, Ayos, Cameroon); A. Ambani, M. Atangana, J-C. Biaback, M. Kennedy, H. Kibedou, F. Kounga, M. Maguip Abanda, E. Mamang, A. Mikone, S. Tang, E. Tchuangue, S. Tchuenko, D. Yakan (District Hospital, Bafia, Cameroon); J. Assandje, S. Ebana, D. Ebo’o, D. Etoundi, G. Ngama, P. Mbarga Ango, J. Mbezele, G. Mbong, C. Moung, N. Ekotto, G. Nguemba Balla, G. Ottou, M. Tigougmo (District Hospital, Mbalmayo, Cameroon); R. Beyala, B. Ebene, C. Effemba, F. Eyebe, M-M. Hadjaratou, T. Mbarga, M. Metou, M. Ndam, B. Ngoa, EB. Ngock, N. Obam (District hospital, Mfou, Cameroon); A. M. Abomo, G. Angoula, E. Ekassi, Essama, J.J. Lentchou, I. Mvilongo, J. Ngapou, F. Ntokombo, V. Ondoua, R. Palawo, S. Sebe, E. Sinou, D. Wankam, I. Zobo (District hospital, Monatélé, Cameroon); B. Akono, A. L. Ambani, L. Bilock, R. Bilo’o, J. Boombhi, F.X. Fouda, M. Guitonga, R. Mad’aa, D.R. Metou’ou, S. Mgbih, A. Noah, M. Tadena, Ntcham (District hospital, Nanga Eboko, Cameroon); G. Ambassa Elime, A.A. Bonongnaba, E. Foaleng, R.M. Heles, R. Messina, O. Nana Ndankou, S.A. Ngono, D. Ngono Menounga, S.S. Sil, L. Tchouamou, B. Zambou (District hospital, Ndikinimeki, Cameroon); R. Abomo, J. Ambomo, C. Beyomo, P. Eloundou, C. Ewole, Joël Fokom Domgue, M. Mvoto, M. Ngadena, R. Nyolo, C. Onana, A. Oyie (District hospital, Obala, Cameroon); P. Antyimi, S. Bella Mbatonga, M. Bikomo, Y. Molo Bodo, S. Ndi Ntang, P. Ndoudoumou, L. Ndzomo, S.O. Ngolo, M. Nkengue, Nkoa, Y. Tchinda (District hospital, Sa’a, Cameroon).
